# Suppression of interleukin-6 increases enterovirus A71 lethality in mice

**DOI:** 10.1186/s12929-017-0401-5

**Published:** 2017-12-12

**Authors:** Li-Chiu Wang, Hui-Wen Yao, Chuan-Fa Chang, Shainn-Wei Wang, Shih-Min Wang, Shun-Hua Chen

**Affiliations:** 1 Institute of Basic Medical Sciences, National Cheng Kung University, Tainan, Taiwan 701 Republic of China; 2 Center of Infectious Disease and Signaling Research, National Cheng Kung University, Tainan, Taiwan 701 Republic of China; 3 Department of Medical Laboratory Science and Biotechnology, National Cheng Kung University, Tainan, Taiwan 701 Republic of China; 4 Institute of Molecular Medicine, National Cheng Kung University, Tainan, Taiwan 701 Republic of China; 5 Department of Pediatrics, National Cheng Kung University, Tainan, Taiwan 701 Republic of China; 6 Department of Microbiology and Immunology, College of Medicine, National Cheng Kung University, Tainan, Taiwan 701 Republic of China

**Keywords:** Enterovirus A71 and interleukin-6

## Abstract

**Background:**

Enterovirus A71 (EV-A71) infection can induce fatal encephalitis in young children. Clinical reports show that interleukin-6 (IL-6) levels in the serum and cerebrospinal fluid of infected patients with brainstem encephalitis are significantly elevated. We used a murine model to address the significance of endogenous IL-6 in EV-A71 infection.

**Results:**

EV-A71 infection transiently increased serum and brain IL-6 protein levels in mice. Most importantly, absence of IL-6 due to gene knockout or depletion of IL-6 using neutralizing monoclonal antibody enhanced the mortality and tissue viral load of infected mice. Absence of IL-6 increased the damage in the central nervous system and decreased the lymphocyte and virus-specific antibody responses of infected mice.

**Conclusions:**

Endogenous IL-6 functions to clear virus and protect the host from EV-A71 infection. Our study raises caution over the use of anti-IL-6 antibody or pentoxifylline to reduce IL-6 for patient treatment.

**Electronic supplementary material:**

The online version of this article (10.1186/s12929-017-0401-5) contains supplementary material, which is available to authorized users.

## Background

Enterovirus A71 (EV-A71) is a neurotropic picornavirus transmitted by the fecal-oral route. It can infect the human central nervous system (CNS) to induce neurological manifestations, such as aseptic meningitis, encephalomyelitis, and brainstem encephalitis, especially in young children [1–4]. Severe symptoms, brainstem encephalitis combined with pulmonary edema, often cause death or long-term neurological sequelae [1]. Widespread and deadly EV-A71 outbreaks have been frequently reported in the Asia-Pacific region, including Taiwan, for two decades [2–5]. Although EV-A71 is becoming an important pathogen for children, its pathogenesis remains elusive.

Previous clinical studies showed that the levels of serum cytokines, including interleukin (IL)-6, IL-1β, IL-10, IL-13, and tumor necrosis factor alpha (TNF-α), in EV-A71-infected patients with both brainstem encephalitis and pulmonary edema were significantly higher than those in patients with only brainstem encephalitis [6–8]. Therefore, a “cytokine storm” has been proposed to trigger cardiopulmonary collapse [6]. Immune suppressors, steroids and intravenous immunoglobulin, have been used or suggested to treat patients with brainstem encephalitis [9–11]. Pentoxifylline, which reduces the production of cytokines like IL-6, is also recommended for patient treatment [6, 12]. Among the high levels of proinflammatory cytokines detected in sera of patients with brainstem encephalitis, IL-6 (with a level of >70 pg/ml) was recognized as the best predictor for developing fatal pulmonary edema complication when compared with IL-1β and TNF-α [6]. High levels of IL-6 have also been detected in the cerebrospinal fluid of patients with CNS involvement [7, 13].

IL-6 is a pleiotropic cytokine secreted by a variety of cells in response to various stimuli, such as infections and other cytokines, including IL-1β, interferon gamma (IFN-γ), and TNF-α [14, 15]. It is a B cell growth and differentiation factor and an inducer of antibody production [14, 16]. It also stimulates the proliferation, differentiation, as well as activation of CD8 T cells and especially, CD4 T cells [16–18]. In the CNS, it is a neurotrophic factor secreted by neurons and glial cells to promote neuronal survival, nerve regeneration, and glial cell activation [19–22]. In humans, the serum IL-6 level has been shown to be inversely associated with the duration of hospitalization and treatment in patients infected with respiratory syncytial virus [23]. However, IL-6 levels in the serum or CNS are considered to be a marker of poor outcomes including death and neurological sequelae in patients with encephalopathy caused by influenza A virus or Japanese encephalitis virus infection [24–28]. In mice, endogenous IL-6 is believed to worsen the diseases induced by influenza A virus or murine cytomegalovirus [29, 30]. On the other hand, endogenous IL-6 is shown to play a protective role in herpes simplex virus, vaccinia virus, or Theiler’s murine encephalomyelitis virus infection [21, 31, 32]. These studies show the controversial roles of IL-6 in viral infections.

After infection of mice with EV-A71, Khong et al. found increasingly elevated serum and tissue IL-6 levels from days 4 to 10 post-infection (p.i.) [33]. Khong et al. assessed the role of IL-6 using antibody to deplete the cytokine at the time of infection. Surprisingly, anti-IL-6 antibody treatment enhanced serum IL-6 levels from days 1 to 4 p.i. Moreover, the results showing that anti-IL-6 antibody treatment decreased (intestine) virus loads but increased the lethality of infected mice are contradictory. To address the significance of endogenous IL-6 in EV-A71 infection, we used two approaches by comparing infected *IL-6* gene knockout mice and wild-type mice and by applying neutralizing monoclonal antibody to deplete IL-6 in wild-type mice. We found that endogenous IL-6 reduced the tissue viral load and mortality of infected mice.

## Methods

### Cells, virus, infection of mice, and anti-IL-6 antibody treatment

The human muscle rhabdomyosarcoma (RD) cell line was maintained and propagated according to the instructions of American Type Culture Collection. EV-A71 strains M2 were propagated and titrated on RD cell monolayers as previously described [34]. All mouse experiment protocols were approved by the Institutional Animal Care and Use Committee of National Cheng Kung University. Fourteen-day-old wild-type C57BL/6J mice and C57BL/6J–derived *IL-6* ^−/−^ (B6.129S2-*Il6* ^*tm1kopf*^/J) mice purchased from the Jackson Laboratory were infected with 3 × 10^5^ plaque forming units (PFU)/mouse of M2 by intraperitoneal inoculation. In addition, infected wild-type mice were treated with the monoclonal antibody against mouse IL-6 (clone MP5-20F3, Bio X Cell) or the control antibody (rat immunoglobulin G) by intraperitoneal injection. Infected mice were examined daily for signs of disease and survival for 30 days, and the disease score was graded as follows: 0, healthy; 1, ruffled hair; 2, weakness in hind limbs; 3, paralysis in single hind limb; 4, paralysis in both hind limbs, and 5, death. In separated experiments, mouse blood, tissues, or organs were harvested after infection to measure viral titers by a plaque assay on RD cells.

### ELISA

Mouse blood was collected and processed into serum. In addition, mice were perfused by intracardial injection of ice-cold phosphate buffered saline. Mouse brains were harvested, frozen at −80 °C, and homogenized in 1 ml phosphate buffered saline containing protease inhibitor cocktail (Sigma-Aldrich). The brain homogenates were centrifuged to obtain supernatants. The IL-6 levels in the sera or brain supernatants were measured using an ELISA (R&D Systems) according to the instructions of manufacturer.

### Histological staining

Briefly, tissues were fixed in 10% neutral buffered formalin, embedded in paraffin, and sectioned. Sections were deparaffinized and stained with hematoxylin and eosin.

### Titration of virus-specific, neutralizing antibodies

Mouse sera were collected 6 and 10 days after infection to determine neutralizing titers of antibodies. Serial twofold dilutions of heat-inactivated serum were mixed with EV-A71 as previously described [35]. The mixtures of serum and virus (100-fold 50% tissue culture infectious dose) were incubated at 37 °C for 1 h before transfer to RD cell monolayers seeded the day before. The cells were incubated for three more days to observe the cytopathic effect. The highest dilution of serum that protected RD cell monolayers from infection was taken as the neutralizing titer.

### Flow cytometry

Mice infected with virus for 10 days were anesthetized and perfused. Mouse spleens were harvested and homogenized. Splenocytes were treated twice with buffer to lyse red blood cells and washed. In addition, leukocytes in the brain were separated by discontinuous Percoll gradient and washed. Cells were stained with FITC- or PE-conjugated control antibodies or monoclonal antibodies against CD4 (clone GK 1.5, eBioscience), CD8a (clone 53–6.7, eBioscience), CD19 (clone 6D5, eBioscience), CD69 (clone H1.2F3, eBioscience), or CD138 (clone 281–2, BD Biosciences). The stained cells were analyzed by a FACSCalibur (BD Biosciences) and data were analyzed by WinMDI software.

### Statistical analyses

Data are expressed as mean + or ± SE values. For statistical comparison, mouse survival rates and the percentages of infected mice producing anti-EV-A71 neutralizing antibody were analyzed by a Fisher’s exact test. Disease scores and tissue viral titers were analyzed by a Mann-Whitney *U* test. Other data were analyzed by a Student’s *t* test. All *P* values were for 2-tailed significance tests. A *P* value of <0.05 is considered statistically significant.

## Results

### EV-A71 infection transiently increases serum and brain IL-6 levels in mice

**Fig. 1:**
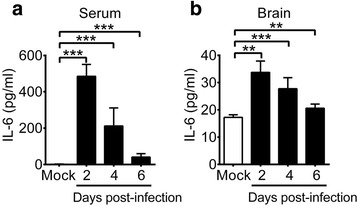
EV-A71 infection increases serum and brain IL-6 levels of mice. IL-6 levels in the sera (**a**) and brains (**b**) of mice, which were mock-infected or infected with EV-A71 for the indicated days, are shown. Data show means + SEM (error bars) of ≥5 samples per group. **P < 0.01 and ***P < 0.001

Fourteen-day-old C57BL/6J mice were mock-infected or infected with 3 × 10^5^ PFU/mouse of EV-A71 strain M2 by intraperitoneal inoculation. Among 19 infected mice, seven displayed signs of encephalitis manifested by hunch posture, ataxia, and hind limb paralysis. The final mortality rate of infected mice was about 37%. We collected mouse sera to measure IL-6 protein using ELISA. In mock-infected mice, the serum IL-6 level was below detection (Fig. 1a). EV-A71 infection significantly increased mouse serum IL-6 levels from days 2 to 6 p.i. (*P* < 0.001). In infected mice, serum IL-6 levels reached a peak (with a concentration of 485 pg/ml) on day 2 p.i. and declined on day 4 p.i. to a very low level (41 pg/ml) on day 6 p.i. We also harvested and homogenized mouse brains to measure IL-6. In the brains of mock-infected mice, IL-6 was constitutively expressed at a level of <20 pg/ml (Fig. 1b). EV-A71 infection significantly increased mouse brain IL-6 levels from days 2 to 6 p.i. (*P* < 0.01). In infected mice, the brain IL-6 level reached a peak and was increased by about 2-fold when compared with that of mock-infected mice on day 2 p.i. The brain IL-6 levels declined on day 4 p.i. and was low (21 pg/ml) on day 6 p.i. These results show that the significant increases of mouse serum and brain IL-6 levels induced by EV-A71 infection were gradually declined from day 4 p.i.

### Absence of IL-6 increases the lethality, tissue damage, and tissue viral loads of infected mice

**Fig. 2:**
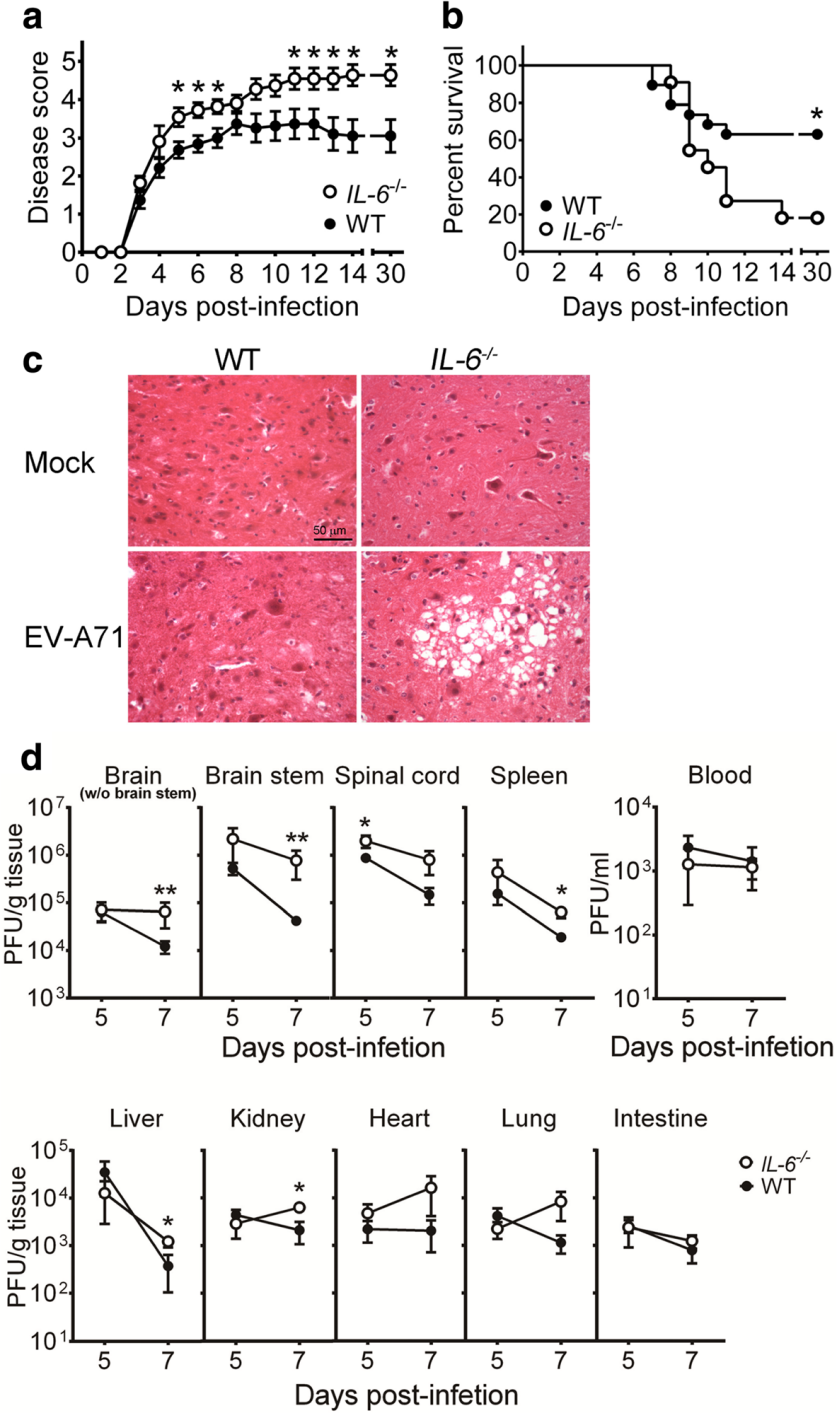
Absence of IL-6 increases the disease severity, mortality, tissue damage, and tissue viral loads of EV-A71-infected mice. The disease severity (**a**) and survival (**b**) of infected wild-type mice (black symbols or WT; *n* = 19) and infected *IL-6* ^−/−^ mice (white symbols; *n* = 11) were monitored for 30 days. (**c**) Spinal cords of mock-infected or infected wild-type and *IL-6* ^−/−^ mice were harvested 10 days after infection, sectioned, and stained with hematoxylin-eosin. The motor horn of lumbar region is shown. Data are representative of at least 3 samples from two independent experiments. (**d**) Viral titers in the indicated tissues of wild-type mice (WT; black circles) and *IL-6* ^−/−^ mice (white circles) collected on 5 or 7 days after infection are shown. Data show means ± SEM (error bars) in panel **a** and 6–7 samples per data point in panel **d**. *P < 0.05 and **P < 0.01 compared between wild-type and *IL-6* ^−/−^ mouse groups at the indicated time

To investigate the role of endogenous IL-6 in EV-A71 infection, we infected and compared wild-type C57BL/6J mice and C57BL/6J–derived mice with a targeted disruption of the gene encoding IL-6. Both wild-type mice and *IL-6* gene knockout (*IL-6* ^−/−^) mice started to display symptoms, weakness in hind limbs and lethargy on day 4 p.i. (Fig. 2a). Overall, the disease score of *IL-6* ^−/−^ mice was significantly higher than that of wild-type mice (*P* < 0.05). Moreover, the final survival rate of *IL-6* ^−/−^ mice (2/9) was significantly lower than that of wild-type mice (12/19) by day 30 p.i. (Fig. 2b, *P* < 0.05). We monitored damage in mouse tissues using hematoxylin-eosin staining. Abundant sponge-like lesions were detected in the motor horns of lumbar spinal cord of infected *IL-6* ^−/−^ mice, but not in infected wild-type mice, on day 10 p.i. (Fig. 2c). The histology result explains severe limb paralysis observed in infected *IL-6* ^−/−^ mice. Viral titers and antigens were below detection in the spinal cord of infected mice on day 10 p.i.

We also monitor tissue viral loads (Fig. 2d). On day 5 p.i., the mean viral titers in the brain stems, spinal cords, spleens, and hearts of *IL-6* ^−/−^ mice were higher than those of wild-type mice with a significant difference found in the spinal cord (*P* < 0.05). On day 7 p.i., the mean viral titers in the CNS regions (brain without the brain stem region, brain stem, and spinal cord) and peripheral organs (spleen, liver, kidney, heart, lung, and intestine) of *IL-6* ^−/−^ mice were all higher than those of wild-type mice by about 3- to 50-fold. The differences in viral titers of several vital organs (the brain without the brain stem region, brain stem, spleen, liver, and kidney) between wild-type and *IL-6* ^−/−^ mouse groups were statistically significant (*P* < 0.05). The viral load results reveal that *IL-6* knockout impairs virus clearance in mouse tissues.

### Absence of IL-6 decreases the levels of virus-specific, neutralizing antibody in the serum and CD4 T cells in the spleen

**Fig. 3:**
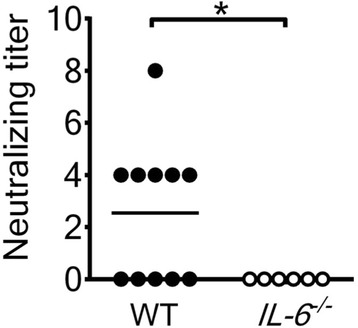
Absence of IL-6 reduces the virus-specific, neutralizing antibody response in the serum of EV-A71-infected mice. The neutralizing antibody titers in sera of wild-type (WT) mice and *IL-6* ^−/−^ mice infected with virus for 10 days are shown. Each symbol on the scatter gram represents an individual sample, and the black horizontal line represents the mean value for wild-type mice. *P < 0.05

Our previous study showed that mice deficient in B cells, CD4 T cells, or CD8 T cells were significantly more susceptible to EV-A71-induced death than wild-type mice with the highest mortality rate found in B cell-deficient mice [36]. Moreover, passive transfer of the neutralizing antibody produced by EV-A71-infected wild-type mice significantly reduced the lethality of EV-A71-infected mice deficient in B cells by decreasing tissue viral loads. These results collectively show the significance of lymphocyte and virus-specific antibody responses in EV-A71-infected mice. IL-6 has been shown to increase antibody production and lymphocyte proliferation [14, 16, 17]. Therefore, we harvested sera from infected mice on days 6 and 10 p.i. to determine neutralizing titers. All sera collected from wild-type and *IL-6* ^−/−^ mice (*n* = 4 in each group) on day 6 p.i. failed to neutralize EV-A71. Among 11 sera collected from infected wild-type mice on day 10 p.i., six were able to neutralize EV-A71 (Fig. 3). However, none of six sera collected from infected *IL-6* ^−/−^ mice on day 10 p.i. were able to neutralize EV-A71. The percentage of infected mice producing neutralizing antibody in the wild-type mouse group is significantly higher than that in the *IL-6* ^−/−^ mouse group (*P* < 0.05). Additionally, the mean neutralizing antibody titer detected in infected wild-type mice is higher than that detected in infected *IL-6* ^−/−^ mice (*P* < 0.05).

**Fig. 4:**
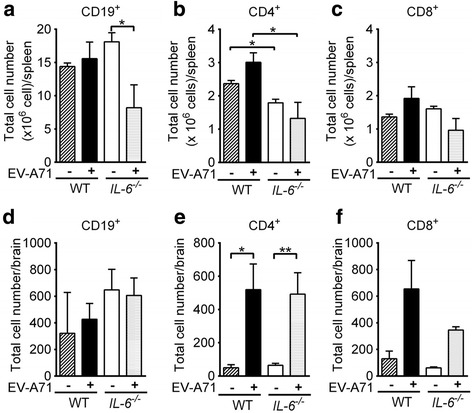
The effect of *IL-6* knockout on tissue lymphocyte numbers in EV-A71-infected mice. The numbers of CD19^+^ cells (**a** and **d**), CD4^+^ cells (**b** and **e**), and CD8^+^ cells (**c** and **f**) in the spleens (**a** to **c**) and brains (**d** to **f**) of wild-type (WT) mice and *IL-6* ^−/−^ mice, which were mock-infected (−) or infected with EV-A71 (+) for 10 days, are shown. Data show means + SEM (error bars) of 5 samples per group. *P < 0.05 and **P < 0.01

To study the effect of IL-6 on the lymphocyte response in tissues, we quantified lymphocytes in the mouse spleen on day 10 p.i. using flow cytometry. In wild-type mice, EV-A71 infection slightly increased the numbers of CD19^+^ B cells, CD4^+^ T cells, and CD8^+^ T cells (Fig. 4a-c). However, in *IL-6* ^−/−^ mice, EV-A71 infection decreased the numbers of all three types of lymphocytes with a significant difference found in the number of CD19^+^ B cells (*P* < 0.05). The mean numbers of CD19^+^ B cells, CD4^+^ T cells, and CD8^+^ T cells in infected wild-type mice were about 2-, 2.3-, and 2-fold, respectively, higher than those of infected *IL-6* ^−/−^ mice with a significant difference found in the number of CD4^+^ T cells (*P* < 0.05). We also quantified the numbers of activated lymphocytes in the spleens of infected mice on day 10 p.i. The mean numbers of activated (CD19^+^ CD138^+^) B cells, (CD69^+^) CD4^+^ T cells, and (CD69^+^) CD8^+^ T cells in infected wild-type mice were about 1.7-, 1.3-, and 1.7-fold, respectively, higher than those of infected *IL-6* ^−/−^ mice (data not shown).

In addition, we quantified lymphocytes in the mouse brain on day 10 p.i. (Fig. 4d-f). In wild-type mice, EV-A71 infection increased the numbers of CD19^+^ B cells, CD4^+^ T cells, and CD8^+^ T cells with a significant difference found in the number of CD4^+^ T cells (*P* < 0.05). In *IL-6* ^−/−^ mice, EV-A71 infection increased the numbers of CD4^+^ T cells and CD8^+^ T cells, but not the number of CD19^+^ B cells. Absence of IL-6 slightly reduced the number of CD8^+^ T cells by about 2-fold and slightly increased the number of CD19^+^ B cells by about 1.4-fold, but failed to affect the number of CD4^+^ T cells, in infected mice. Furthermore, the mean numbers of activated (CD69^+^) CD4^+^ and CD8^+^ T cells in infected wild-type mice were 2.4- and 2.9-fold, respectively, higher than those infected *IL-6* ^*−/−*^ mice (in Additional file 1: Figure S1).

### Anti-IL-6 antibody treatment fails to protect wild-type mice from infection

**Fig. 5:**
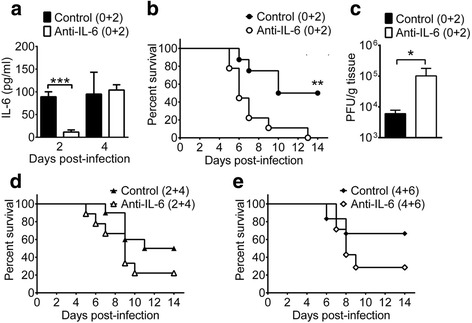
The effects of anti-IL-6 antibody treatment on the serum IL-6 level, survival rate, and tissue viral load of infected wild-type mice. Infected wild-type mice were treated with control or anti-IL-6 antibody on days 0 and 2 after infection and monitored for serum IL-6 levels (**a**), survival rates (**b**), and day-6 lung viral loads (**c**). Infected wild-type mice were treated with antibody on days 2 and 4 after infection (**d**) or on days 4 and 6 after infection (**e**) and monitored for survival rates. Data show means + SEM (error bars) of 3–4 samples per group in panel **a**, 8–9 mice per group in panel **b**, 5–6 samples per group in panel **c**, 9–10 mice per group in panel **d**, and 6–7 mice per group in panel **e**. *P < 0.05, **P < 0.01, and ***P < 0.001 compared between indicated groups or wild-type and IL-6^−/−^ mouse groups

To further confirm the significance of endogenous IL-6 in EV-A71 infection using another approach, we tested to deplete IL-6 in infected wild-type mice using neutralizing monoclonal antibody. Mice were treated with one shot of control or anti-IL-6 antibody at the doses of 4.5, 9, or 18 mg/kg on the day of infection. Anti-IL-6 antibody treatment failed to significantly reduce the survival of infected mice when compared with the control antibody regardless the antibody dose used for treatment (data not shown). We then assessed to give mice antibody on two days (days 0 and 2) after infection at the dose of 4.5 mg/kg in one shot. Compared with the control antibody (Fig. 5a), anti-IL-6 antibody treatment significantly decreased the serum IL-6 protein level on day 2 p.i. (*P* < 0.001), but failed to significantly reduce the brain IL-6 protein levels on days 2 and 4 p.i. (data now shown). As shown in Fig. 5b, the survival rate of infected mice treated with anti-IL-6 antibody (0/9) was significantly lower than that of infected mice treated with control antibody (4/8). Treatment with anti-IL-6 antibody significantly increased the viral load in the mouse lung (*P* < 0.05) by more than one log (Fig. 5c) and slightly increased the viral load in all other mouse tissues (heart, spleen, liver, kidney, brain without brain stem, brain stem, and spinal cord) examined on day 6 p.i. (in Additional file 1: Figure S2), when compared with the control antibody. We also tested to treat mice with antibody at later time points. The survival rates of infected mice treated with anti-IL-6 or control antibody on days 2 and 4 p.i. were 2/9 and 5/10, respectively (Fig. 5d). The survival rates of infected mice treated with anti-IL-6 or control antibody on days 4 and 6 p.i. were 2/7 and 4/6, respectively (Fig. 5e).

## Discussion

Here we show that EV-A71 infection transiently increases mouse serum and brain IL-6 levels, which reach peaks on day 2 p.i. and then decline afterward. More importantly, by comparing infected *IL-6* ^*−/−*^ and wild-type mice and by depleting IL-6 in infected wild-type mice using a neutralizing monoclonal antibody, we uncover that endogenous IL-6 functions to protect mice from EV-A71 infection and reduce tissue viral loads. Absence of IL-6 decreases levels of virus-specific, neutralizing antibody in the serum and CD4 T cells in the spleen. Previous mouse and clinic studies recommended to use anti-IL-6 antibody or pentoxifylline to reduce IL-6 for patient treatment [6, 33]. Our present study raises caution over such practice.

Khong et al. observed increasingly elevated IL-6 levels in tissues, especially in the serum 4 to 10 days after infection of one-day-old BALB/c mice with a clinical isolate, which induced a mortality rate of 75–90% [33]. However, we found transiently elevated serum and brain IL-6 levels, which reached peaks on day 2 p.i. and gradually declined from day 4 p.i., in 14-day-old C57BL/6J mice infected with 3 × 10^5^ PFU/mouse of EV-A71 strain M2, which has been adapted in mice [34] and induced a mortality rate of 37%. Our additional study using 1 × 10^5^ or 1 × 10^6^ PFU/mouse of M2 to infect 14-day-old C57BL/6J mice, which resulted in mortality rates of 0 and 100%, also found transiently elevated serum IL-6 levels, which declined from day 4 p.i. to basal levels (< 45 pg/ml) on day 6 p.i., in both groups. We also tested to infect 1- to 3-day-old BALB/c mice with 100 PFU/mouse of M2, which induced a mortality rate of 100% in mice, and observed that serum IL-6 levels were gradually declined from day 6 p.i. to a basal level on day 8 p.i. (in Additional file 1: Figure S3). Similar results were obtained in 1- to 3-day-old BALB/c mice infected with a clinical isolate, strain 4643, which is the parental strain of M2 and induced a mortality rate of 44% in mice at the dose of 8 × 10^6^ PFU/mouse (in Additional file 1: Figure S3). Collectively, we found transiently, but not increasingly, elevated serum IL-6 levels using two virus strains, various viral doses, and two backgrounds of mice at different ages with mortality rates of infected mice ranging 0–100%. In patients and mice, EV-A71 infection enhances the expression of IL-1β, IFN-γ, and TNF-α, which can induce IL-6 expression [6, 7, 13, 37, 38]. In addition, our study using 1 × 10^5^ or 3 × 10^5^ PFU/mouse of M2 to infect 14-day-old C57BL/6J mice showed that the viral doses, mouse mortality rates (0 and 37%), and peak serum IL-6 levels (<100 and 485 pg/ml) were positively correlated. Further studies are needed to identify the IL-6 producer and inducer and to determine whether virus or viral protein(s) may serve as an IL-6 inducer.

Khong et al. assessed the role of IL-6 in EV-A71 infection using monoclonal antibody to deplete the cytokine [33]. Infection of one-day-old BALB/c mice and treatment with anti-IL-6 antibody at the time of infection reduced the viral load in the tissue (intestine) from days 1 to 7 p.i. but increased the mouse mortality rate. Treatment with anti-IL-6 antibody on day 3 or 6 p.i. failed to reduce viral titers in the tissue (intestine), but decreased mouse mortality rates with diminished damage in muscle. They concluded that anti-IL-6 antibody treatment represents a potential therapeutic approach and that the protection mediated by anti-IL-6 antibody is independent of viral load. We also utilized the same monoclonal antibody to deplete IL-6 in infected wild-type mice. Treatment with anti-IL-6 antibody on days 0 and 2 p.i. significantly reduced the survival rate of infected mice by 50%. Treatment with anti-IL-6 antibody on days 2 and 4 or days 4 and 6 p.i. slightly reduced the survival rates of infected mice by 28% and 38%, respectively.

In mice, not only the roles of IL-6 in viral infections are controversial, but the protective mechanisms mediated by IL-6 also vary. Endogenous IL-6 reduces the tissue viral loads of mice infected with a virulent vaccinia virus through cytotoxic T-cell response [32]. It reduces the mortality of Theiler’s murine encephalomyelitis virus-infected mice by decreasing neuronal damage, but not by decreasing viral loads [21]. However, how IL-6 improves the survival of herpes simplex virus-infected mice and why it fails to affect the cytotoxic T cell response against lymphocytic choriomeningitis virus or an attenuated vaccinia virus remain unclear [31, 32]. Here we show that absence of IL-6 reduces EV-A71 lethality, the damage in the CNS, and tissue viral loads with increased levels of CD4 T cells in the spleen and virus-specific neutralizing antibody in the serum in a manner rarely reported in other virus infections. Very few studies address the effect of endogenous IL-6 on the production of virus-specific, neutralizing antibody. Reports showed that IL-6 deficiency reduces the humoral response in mice infected with murine leukemia virus, but not in mice infected with Theiler’s murine encephalomyelitis virus [21, 39]. Our additional results showed that absence of IL-6 transiently reduced the serum IL-1β protein level on day 2 p,i., but failed to affect brain IL-1β and TNF-α protein levels as well as serum TNF-α protein levels of infected mice on both days 2 and 4 p.i. (in Additional file 1: Figure S4).

Our result shows that absence of IL-6 significantly decreases the number of CD4 T cells in the spleen of infected mice. IL-6 has been shown to increase T cell proliferation by preventing apoptosis [18]. However, we found that the numbers of dead (7-Amino-actinomycin D^+^) cells in the spleens of infected wild-type and *IL-6* ^−/−^ mice were not significant different on day 10 p.i. (data not shown), suggesting that IL-6 increases T cell proliferation in a mechanism independent of cell death in our model. In addition to the capacity of increasing the T cell number, IL-6 has also been reported to enhance T cell response by reducing regulatory T cells, which down regulates IL-17-secreting T helper cells [40, 41]. However, we found that the number of (Foxp3^+^ CD4^+^) regulatory T cells in the spleens of wild-type mice were higher than that of *IL-6* ^−/−^ mice by 10-fold on day 10 p.i. (in Additional file 1: Figure S5). IL-6 is shown to enhance the growth of CD4 T cells [16–18]. In EV-A71-infected mice, high levels of serum IL-6 might stimulate the proliferation of CD4 T cells in the spleen, which may result in the presence of virus-specific, neutralizing antibody in infected wild-type mice, but not in infected *IL-6* ^−/−^ mice, on day 10 p.i.

EV-A71 infection has become a major threat to the public health in the Asia-Pacific region [4, 5], but there are no effective therapies to control fatal outbreaks. The excessive cytokine response found in patients with fatal symptoms has led to the recommendation of using pentoxifylline, which reduces IL-6 production, or the use of corticosteroids to treat severe cases [6, 10–12]. However, our present study and our previous reports using lymphocyte-deficient mice or corticosteroids to treat mice showed that immunocompromised mice are highly susceptible to EV-A71 infection with increased tissue viral loads [36, 42]. These results refute the use of anti-IL-6 antibody, pentoxifylline, and corticosteroids for patient treatment. The issue regarding whether IL-6 can be used for treatment needs further evaluation, as a report showed that combined IL-6, IL-13, and IFN-γ treatment provokes pulmonary abnormality in EV-A71-infected mice [43]. Overall, our results demonstrate that the tissue viral load and mortality in infected mice are positively correlated, suggesting that early inhibition of viral replication using vaccines or passive transfer of virus-specific, neutralizing antibody should be promising in promoting host survival during EV-A71 infection.

## Conclusion

The present study found that endogenous IL-6 functions to reduce tissue viral loads, enhance the adaptive immunity, and decrease the mortality of EV-A71-infected mice. These results raise caution over the use of anti-IL-6 antibody or pentoxifylline to reduce IL-6 for patient treatment.

## Additional file

Additional file 1
**Figure S1.** Effects of *IL-6* knockout on numbers of activated T cells in infected mouse brains. The numbers of activated (CD69^+^) CD4^+^ and (CD69^+^) CD8^+^ T cells in the brain of wild-type (WT) and *IL-6* ^*−/−*^ mice, which were mock-infected (−) or infected with EV-A71 (+) for 10 days, are shown. Data show means + SEM (error bars) of 3 samples per group. **P* < 0.05 and ***P* < 0.01. **Figure S2.** Effects of anti-IL-6 antibody treatment on tissue viral loads of infected wild-type mice. Tissues of C57BL/6J mice treated with control antibody (*n* = 6) or anti-IL-6 antibody (*n* = 5) on days 0 and 2 after infection were collected on day 6 after infection to determine viral titers. Data show means + SEM (error bars). **Figure S3.** Gradually declined serum IL-6 levels in infected BALB/c mice from day 4 post-infection. One- to three-day mice were mock-infected (M) or infected with EV-A71 strain M2 (**a**) or strain 4643 (**b**). The serum IL-6 levels are shown. Data show means + SEM (error bars) of ≥3 samples per group. **P* < 0.05 and ****P* < 0.001. **Figure S4.** Effects of *IL-6* knockout on cytokine levels in infected mice. IL-1β and TNF-α levels in the serum (**a** and **b**) and brain (**c** and **d**) of mock-infected (M) mice (*n* = 3–4/group) and infected wild-type (WT) mice (*n* = 6/group) and *IL-6* ^−/−^ mice (*n* = 6/group) are shown. Data show means + SEM. **P* < 0.05. **Figure S5.** Effects of *IL-6* knockout on numbers of regulatory T cells in the spleen of infected mice. The numbers of CD4^+^ Foxp3^+^ splenocytes in wild-type (WT) mice and *IL-6* ^−/−^ mice, which were mock-infected (−) or infected with EV-A71 (+) for 10 days, are shown. Data show means + SEM (error bars) of 3 samples per group. (DOC 7204 kb)

## Abbreviations

EV-A71: enterovirus A71
IL-6: interleukin-6
p.i.: post-infection
PFU: plaque forming units
